# Conjugated polyelectrolyte hole transport layer for inverted-type perovskite solar cells

**DOI:** 10.1038/ncomms8348

**Published:** 2015-06-17

**Authors:** Hyosung Choi, Cheng-Kang Mai, Hak-Beom Kim, Jaeki Jeong, Seyeong Song, Guillermo C. Bazan, Jin Young Kim, Alan J. Heeger

**Affiliations:** 1 Department of Chemistry, College of Natural Sciences, Hanyang University, Seoul 133-791, Republic of Korea; 2 Center for Polymers and Organic Solids, University of California Santa Barbara, Santa Barbara, California 93106, USA; 3 School of Energy and Chemical Engineering, Ulsan National Institute of Science and Technology (UNIST), Ulsan 689-798, Republic of Korea

## Abstract

Organic–inorganic hybrid perovskite materials offer the potential for realization of low-cost and flexible next-generation solar cells fabricated by low-temperature solution processing. Although efficiencies of perovskite solar cells have dramatically improved up to 19% within the past 5 years, there is still considerable room for further improvement in device efficiency and stability through development of novel materials and device architectures. Here we demonstrate that inverted-type perovskite solar cells with pH-neutral and low-temperature solution-processable conjugated polyelectrolyte as the hole transport layer (instead of acidic PEDOT:PSS) exhibit a device efficiency of over 12% and improved device stability in air. As an alternative to PEDOT:PSS, this work is the first report on the use of an organic hole transport material that enables the formation of uniform perovskite films with complete surface coverage and the demonstration of efficient, stable perovskite/fullerene planar heterojunction solar cells.

## Abstract (web summary)

There is intensive research underway into the development of hole transport layers for perovskite solar cells. Here, the authors report an inverted-type perovskite solar cell with low-temperature solution-processable conjugated polyelectrolyte as the hole transport layer, which exhibits an efficiency of over 12%.

## Graphical abstract

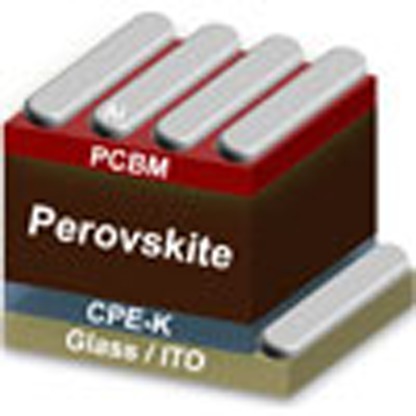

Organic–inorganic hybrid perovskites have attracted considerable attention as possible next-generation thin-film solar cells because of advantages such as the low-cost of precursors, easy tuning of the bandgap, broad light absorption throughout the visible wavelength region, long exciton diffusion length and solution processability^1^,^2^,^3^,^4^,^5^. Efforts dedicated towards improving device architectures and optimization of perovskite film morphology have improved power conversion efficiencies (PCEs) of perovskite solar cells (pero-SCs) up to 19% (refs 6, 7, 8, 9, 10, 11). The fabrication of conventional pero-SCs, however, high-temperature annealing is required for the metal oxides (titanium oxide and zinc oxide are commonly used as the electron transport layer). The high-temperature annealing is not consistent with the use of flexible plastic substrates.

Inverted-type pero-SCs (ipero-SCs) have emerged as an alternative to conventional pero-SCs because of their low-temperature solution processability. In this strategy, poly(3,4-ethylenedioxythiophene):poly-styrene sulfonate (PEDOT:PSS) is used as the hole transport layer (HTL). However, there is evidence that the acidic nature of PEDOT:PSS is detrimental to long-term device performance and stability^12^. As an alternative to PEDOT:PSS, inorganic HTLs previously developed for polymer solar cells have been introduced into ipero-SCs. Docampo *et al.* reported that hole transport through vanadium oxide (V_2_O_5_) and nickel oxide (NiO) are comparable to that of PEDOT:PSS^13^. However, ipero-SCs based on inorganic HTLs and methylammonium lead mixed halide (MAPbI_3-X_Cl_X_)/[6,6]-phenyl-C_61_ butyric acid methyl ester (PCBM) planar heterojunction structures exhibited poor device performance because of incomplete surface coverage as a result of different surface energies of perovskite solution and V_2_O_5_ or NiO substrates. Recently, ipero-SCs based on methylammonium lead iodide (MAPbI_3_) achieved PCEs of ∼9.5% by using mesoporous NiO as the HTL^14^. However, thermal annealing treatment over 400 °C was needed to convert amorphous NiO into nanocrystalline NiO films. Moreover, the PCEs were still lower than those of the devices with PEDOT:PSS. It is therefore necessary to find new HTLs that are compatible with perovskite precursor solution and low-temperature solution processing. To the best of our knowledge, there have been few reports on high-performance ipero-SCs using organic HTLs instead of PEDOT:PSS and p-type metal oxides^10^,^15^,^16^.

Here we report ipero-SCs that take advantage of a novel pH-neutral and low-temperature solution-processable conjugated polyelectrolyte (CPE) as the HTL. Among various CPEs (Supplementary Table 1), we employ a poly[2,6-(4,4-bis-potassiumbutanylsulfonate-4H-cyclopenta-[2,1-b;3,4-b']-dithiophene)-alt-4,7-(2,1,3-benzothiadiazole)] (CPE-K) because CPE-K results in highest PCE (Supplementary Fig. 1 and Supplementary Table 2), which was previously used in polymer solar cells^17^. These devices with CPE-K achieve a PCE of over 12% with enhanced device stability under ambient conditions. These improvements are attributed to the excellent wetting of perovskite precursor solution on the CPE layer, efficient hole selectivity between the perovskite and indium tin oxide (ITO) anode and pH-neutral CPE-K solution.

## Results

### Optical properties

**Figure 1:**
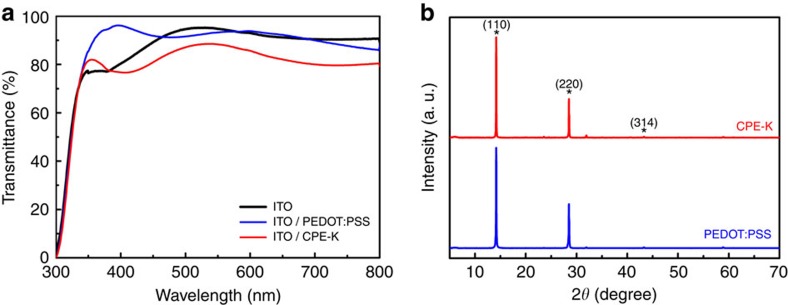
Effect of PEDOT:PSS and CPE-K on optical property and perovskite crystallinity. (**a**) Comparison of transmittance between PEDOT:PSS and CPE-K on the ITO substrate. (**b**) XRD patterns of perovskite films on PEDOT:PSS and CPE-K layer.

We first compared the transmittance of PEDOT:PSS and CPE-K and absorption of perovskite films coated on top of them. Figure 1a provides the transmittance spectra of PEDOT:PSS and CPE-K films spin-coated on ITO substrates. Bare ITO is also included for comparison. Compared with PEDOT:PSS, CPE-K showed lower transmittance in the range of 350–500 and 600–850 nm because of its narrow bandgap (1.4 eV)^18^. Before depositing perovskite films on different substrates, we tested whether the CPE-K film is washed out by the solvent used for perovskite precursor deposition, namely *N,N*-dimethylformamide (DMF). Although the absorption of the CPE-K film was slightly reduced after spin-coating from DMF (Supplementary Fig. 2), the perovskite film can be deposited on CPE-K without complete removal of the underlayer, as confirmed by the absorption spectra of MAPbI_3−*X*_Cl_*X*_ perovskite films spin-coated on PEDOT:PSS and CPE-K (Supplementary Fig. 3). Perovskite films on CPE-K exhibited slightly higher optical density than perovskite on PEDOT:PSS in the range of 500–850 nm because of absorption of CPE-K (Inset of Supplementary Fig. 2). Regardless of substrates, both perovskite films with the thickness of 250±20 nm exhibited broad and high light absorption in the visible wavelength region.

### Film morphology

To investigate the influence of PEDOT:PSS and CPE-K on perovskite crystallinity, we performed X-ray diffraction (XRD) measurement. We prepared perovskite films on top of glass substrates coated with PEDOT:PSS and CPE-K. Both films exhibited diffraction peaks at 14.15°, 28.47° and 43.12°, corresponding to (110), (220) and (314) planes of the tetragonal perovskite phase (Fig. 1b). These peaks are consistent with XRD results in previous reports on pero-SCs^3^,^5^,^19^. There were negligible differences in the intensity of diffraction peaks between the two underlayers, implying that both PEDOT:PSS and CPE-K are appropriate substrates for transforming perovskite precursor materials into the desirable perovskite crystal phase.

**Figure 2:**
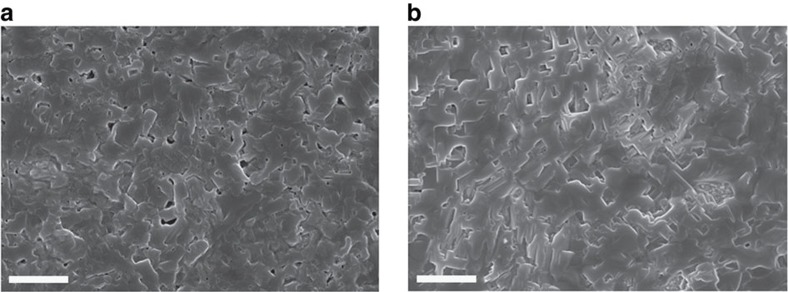
Perovskite film morphology. SEM top-view images of perovskite films spin-coated on top of (**a**) PEDOT:PSS and (**b**) CPE-K. Scale bar, 2 μm.

Surface coverage and the morphology of perovskite film on specific substrates are crucial for determining resultant performance of pero-SCs^6^,^8^. To compare the morphology of perovskite films on PEDOT:PSS and CPE-K, we utilized scanning electron microscopy (SEM) and atomic force microscopy (AFM). Figure 2 presents the top-view SEM images of perovskite films. Spin-coating atop PEDOT:PSS leads to incomplete surface coverage with small voids between crystal boundaries (Fig. 2a), whereas the CPE-K layer provides a uniform perovskite film without voids (Fig. 2b). AFM topography images were consistent with SEM results. Although both PEDOT:PSS and CPE-K layers provided uniform films with root-mean-square (r.m.s.) roughness of 1.0 nm (Supplementary Fig. 4), the perovskite film on CPE-K showed a more even surface with high surface coverage and a roughness of 14.7 nm compared with films deposited on PEDOT:PSS (roughness: 15.6 nm; Supplementary Fig. 5).

We also studied the surface energy of PEDOT:PSS and CPE-K by performing contact angle measurements. Contact angles of PEDOT:PSS and CPE-K films to DMF were extremely low (<3°, Supplementary Fig. 6) and therefore both layers provide excellent wettability with DMF. These results reveal that the physical properties of CPE-K relevant for fabrication of devices, such as wettability and hydrophilicity, are compatible with perovskite solution and enable the formation of high coverage uniform perovskite films.

### Charge transfer dynamics

**Figure 3:**
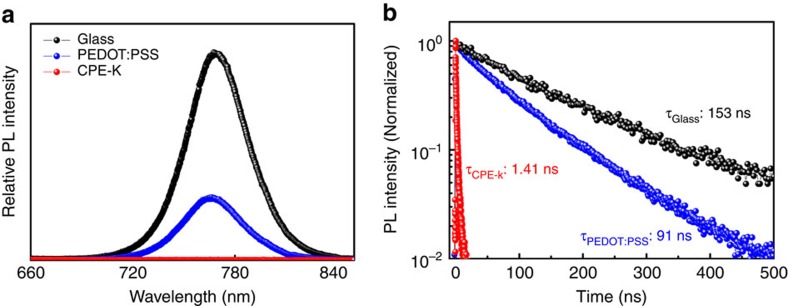
Photoluminescence response of perovskite films on different substrates. (**a**) Steady-state PL spectra and (**b**) time-resolved PL decay transients of perovskite films on different substrates. PL decay transients were collected at 770 nm for all films in vacuum after excitation at 405 nm.

To investigate hole selectivity of the perovskite light absorber to the ITO anode, we carried out photoluminescence (PL) and time-resolved PL decay measurements. We prepared samples with configuration of glass/(PEDOT:PSS or CPE-K)/perovskite, where thick perovskite films (thickness: 250±20 nm) were used for optimum device performance. CPE-K led to perovskite with more efficient PL quenching than PEDOT:PSS, with quenching efficiencies of 71% and 99% for PEDOT:PSS and CPE-K, respectively (Fig. 3a). Figure 3b presents the time-resolved PL decay transients on different substrates. From the calculation of decay values, we obtained average PL decay time (*τ*_aver_) of 153 ns for bare glass/perovskite, 91 ns for glass/PEDOT:PSS/perovskite and 1.41 ns for glass/CPE-K/perovskite. These values are comparable to the PL decay time of MAPbI_3−*X*_Cl_*X*_ perovskite reported previously^2^. Compared with the perovskite on PEDOT:PSS, CPE-K significantly reduced the PL decay time, implying that holes separated from photo-generated excitons within the perovskite layer are efficiently extracted from perovskite to CPE-K. PL quenching and time-resolved PL decay measurements confirm the capability of CPE-K to extract and transport holes from the perovskite layer to the ITO anode. This result is in good agreement with data obtained using CPE-K as the hole extraction layer in polymer solar cells^15^.

### Solar cell performance

**Figure 4:**
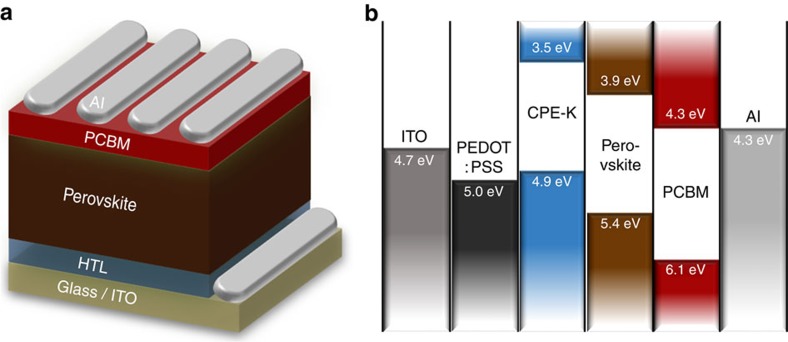
Structure of perovskite solar cells. (**a**) Device architecture and (**b**) energy-band diagram of the devices with PEDOT:PSS and CPE-K as the HTL.

**Figure 5:**
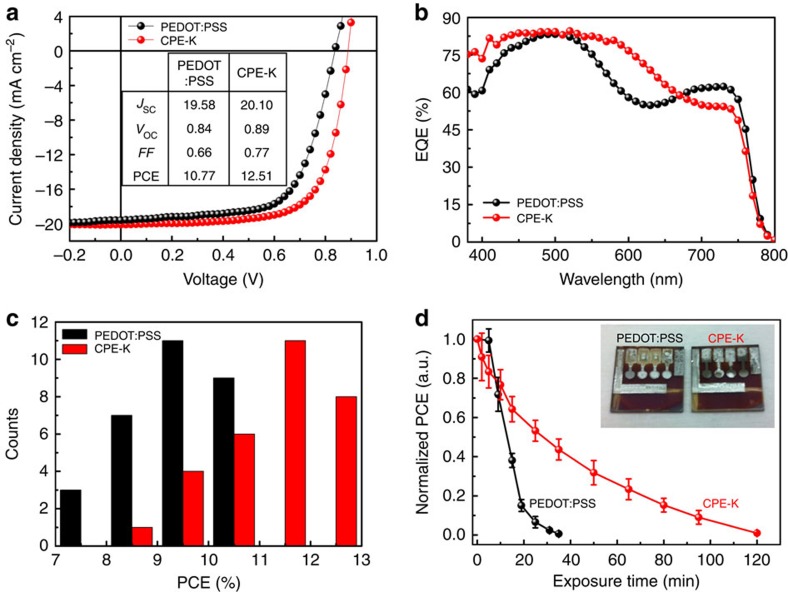
Solar cell performance and stability. (**a**) Current density–voltage (*J–V*) curves, (**b**) EQE, (**c**) efficiency distribution diagram and (**d**) device stability of ipero-SCs with PEDOT:PSS and CPE-K under ambient air condition. Inset table of **a** indicates solar cell parameters, *J*_SC_ (mA cm^−2^), *V*_OC_ (V), FF and PCE (%). Inset images of **d** exhibit the photos of real devices with PEDOT:PSS and CPE-K after air exposure for 12 h.

To verify the merits of CPE-K in devices, we fabricated ipero-SCs using the simple architecture ITO/PEDOT:PSS or CPE-K/MAPbI_3−*X*_Cl_*X*_ perovskite/PCBM/Al (Fig. 4a).The highest occupied molecular orbital (HOMO) and lowest unoccupied molecular orbital (LUMO) levels of CPE-K (HOMO: 4.9 and LUMO: 3.5 eV) are well matched with the valence (VB) and conduction band (CB) of the perovskite (VB: 5.4 and CB: 3.9 eV), respectively, thereby facilitating hole transport and blocking electron transport from perovskite to the ITO anode (Fig. 4b). PCBM was used as the electron transport layer because of the well-known efficient electron transport/hole-blocking capability from perovskite to the Al cathode. Figure 5a presents current density–voltage (*J–V*) curves of best ipero-SCs using PEDOT:PSS and CPE-K as the HTL. Devices with PEDOT:PSS exhibited PCE of 10.77% with short-circuit current density (*J*_SC_) of 19.58 mA cm^−2^, open-circuit voltage (*V*_OC_) of 0.84 V and fill factor (FF) of 0.66. Replacing PEDOT:PSS with CPE-K led to a significant enhancement in device efficiency. The device with CPE-K yielded a PCE of 12.51% with *J*_SC_ of 20.10 mA cm^−2^, *V*_OC_ of 0.89 V and FF of 0.70. The detailed solar cell parameters are listed in the inset table in Fig. 5a. The high *J*_SC_ of the devices with PEDOT:PSS and CPE-K is consistent with calculated *J*_SC_ from external quantum efficiency (EQE) curves (PEDOT:PSS: 18.56 and CPE-K: 19.71 mA cm^−2^; Fig. 5b). The margin of error between *J*_SC_ from *J–V* and EQE measurements was ±5%. Interestingly, the EQE curve shape of the device with CPE-K differed from that of the device with PEDOT:PSS. Compared with EQE values of the device with PEDOT:PSS, EQE values of the device with CPE-K showed higher EQE values in the range of 400–650 nm and lower EQE values in the range of 660–800 nm. This phenomenon may be attributed to distinct differences in light absorption and interference effect between PEDOT:PSS and CPE-K. We also tested hysteresis of the devices with PEDOT:PSS and CPE-K. There was slight hysteresis in both devices that may be attributed to interfacial traps induced in HTL or ferroelectric property of perovskite (Supplementary Fig. 7 and Supplementary Table 3)^20^,^21^.

## Discussion

To confirm the reproducibility of device performance, we tested 30 devices that were fabricated using PEDOT:PSS and CPE-K with optimum thickness. Figure 5c presents a histogram of device efficiencies for ipero-SCs based on PEDOT:PSS and CPE-K. Average PCEs of the devices with CPE-K (11.20%) were higher than those of the device with PEDOT:PSS (9.37%). Devices with either PEDOT:PSS or CPE-K exhibited similar *V*_OC_ (0.84–0.90 V) with high reproducibility, whereas *J*_SC_ and FF of the device with CPE-K were mainly higher than those of the device with PEDOT:PSS (Supplementary Fig. 8). Although we also utilized the simple device structure (ITO/HTL/perovskite/PCBM/Al), the average PCEs of the devices with PEDOT:PSS reported here are comparable to or higher than those of ipero-SCs with similar structure reported in the previous literature (<8%)^19^,^22^,^23^. Furthermore, the PCE>12% of the device with CPE-K is one of the highest values in ipero-SCs with various hole and electron transport materials (NiO_*X*_, TiO_*X*_ and LiF, and so on)^9^,^13^,^14^,^24^,^25^.

We also tested the stability of perovskite films and the devices with different HTL under ambient air condition. Average temperature and humidity were 20±3 °C and 40±10% for testing, respectively. We observed degradation in only perovskite film coated on PEDOT:PSS after exposure to air for 12 h (Supplementary Fig. 9). After 24 h, although the film on CPE-K started to degrade, its degradation rate was slower than that of the film on PEDOT:PSS. After 108 h, the colour of the perovskite film on PEDOT:PSS had changed from dark brown to yellow, whereas the film on CPE-K retained the brown colour. This suggests that the acidic nature of PEDOT:PSS accelerates the degradation of the perovskite film. Figure 5d presents normalized PCEs of ipero-SCs with PEDOT:PSS and CPE-K as a function of air exposure time. As expected from the film stability test, the devices with PEDOT:PSS exhibited a more rapid decrease in device efficiency than the devices with CPE-K. After air exposure for 35 min, the reduction rate of PCEs was 99% for the device with PEDOT:PSS and 55% for the device with CPE-K. Long air exposure times, over 12 h, resulted in severe corrosion of Al electrode caused by decomposition of perovskite film in the device with PEDOT:PSS (Inset of Fig. 5d)^26^,^27^, which further confirms that CPE-K is beneficial for improving device stability.

In summary, we have successfully employed pH-neutral and low-temperature solution-processable CPE-K as the HTL in inverted-type perovskite solar cells. Excellent wetting of perovskite precursor solution on the CPE-K layer leads to uniform active layer film with complete surface coverage and superior hole selectivity for facilitating hole transport from perovskite to the ITO anode. As a result, the device with CPE-K exhibits higher device efficiency, over 12%, than that of the device fabricated with widely used PEDOT:PSS. Furthermore, CPE-K improves the device stability in air because of the neutral pH of the underlayer. As an alternative to PEDOT:PSS and p-type metal oxides, CPE-K is a promising hole transport material for efficient perovskite/fullerene planar heterojunction solar cells that can be used on flexible substrates via roll-to-roll processing. This strategy also offers a new approach to design hybrid tandem solar cells employing CPE-K as the intermediate layer, and combining organometallic perovskites and small bandgap organic semiconductors as the active layer.

## Methods

### Solar cell fabrication and characterization

ITO-coated glass substrates were cleaned using sequential ultrasonication in deionized water, acetone and isopropanol for 10 min each. A poly(3,4-ethylenedioxythiophene):polystyrene sulfonate (PEDOT:PSS, Clevios P VP AI 4083, Heraeus) was spin-cast at 5,000 r.p.m. on ultraviolet ozone-treated ITO substrates and dried at 140 °C for 10 min. We prepared CPE-K by following synthetic routes in previous reports^18^,^28^. For the CPE-K layer (thickness: 10 nm), we spin-cast CPE-K solution with concentration of 0.25 wt.% in solvent mixture of deionized water and methanol (1:1 vol.%) and the film was dried at 80 °C for 10 min. After transferring samples into nitrogen-filled glovebox, precursor solutions of MAPbI_3−*X*_Cl_*X*_ perovskite were spin-cast at 7,000 r.p.m. on top of PEDOT:PSS and CPE-K layer, and baked at 90 °C for 60 min. A PCBM solution with a concentration of 1.3 wt.% in chloroform was spin-cast at 3,000 r.p.m. on top of the perovskite layer. Subsequently, an Al electrode with thickness of 100 nm was deposited on top of the PCBM under vacuum (<10^−6 ^Torr) by thermal evaporation. The area of the Al electrode defines the active area of the device as 3.30 mm^2^. The *J–V* characteristics of the solar cells were measured by a Keithley 2400 Source Measure Unit. The solar cell performance was tested with an Air Mass 1.5 Global (AM 1.5 G) solar simulator with an irradiation intensity of 100 mW cm^−2^. EQE measurements were obtained using the PV measurement QE system by applying monochromatic light from a xenon lamp under ambient conditions. The monochromatic light intensity was calibrated using a Si photodiode and chopped at 100 Hz. Masks (1.70 mm^2^) made of thin black plastic were attached to each cell before measurement of the *J–V* characteristics and the EQE to accurately measure the performance of solar cells. All devices were tested in ambient air after ultraviolet epoxy encapsulation.

## Additional information

**How to cite this article:** Choi, H. *et al.* Conjugated polyelectrolyte hole transport layer for inverted-type perovskite solar cells. *Nat. Commun.* 6:7348 doi: 10.1038/ncomms8348 (2015).

## Supplementary Material

Supplementary Information Supplementary Figures 1-9, Supplementary Tables 1-3, Supplementary Methods and Supplementary References
