# Supplementary material for: Conjugated polyelectrolyte hole transport layer for inverted-type perovskite solar cells
Source: Nat Commun. 2015 Jun 17;6:7348. doi: 10.1038/ncomms8348 (PMC4557298; doi:10.1038/ncomms8348)
Supplement: Supplementary Information — Supplementary Figures 1-9, Supplementary Tables 1-3, Supplementary Methods and Supplementary References [file ncomms8348-s1.pdf]

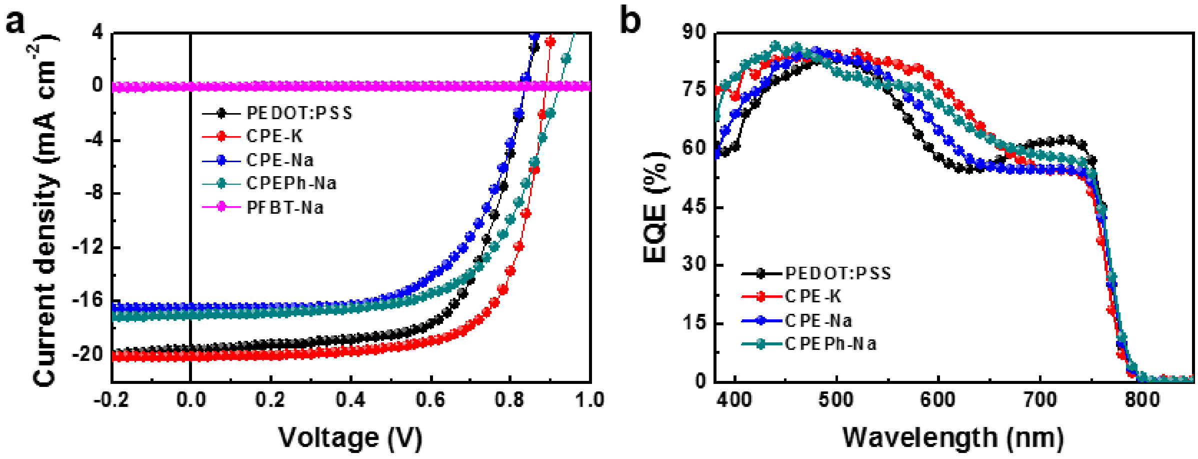

Supplementary Fig. 1. (a) *J*-*V* curves and (b) EQE of ipero-SCs with different HTLs.

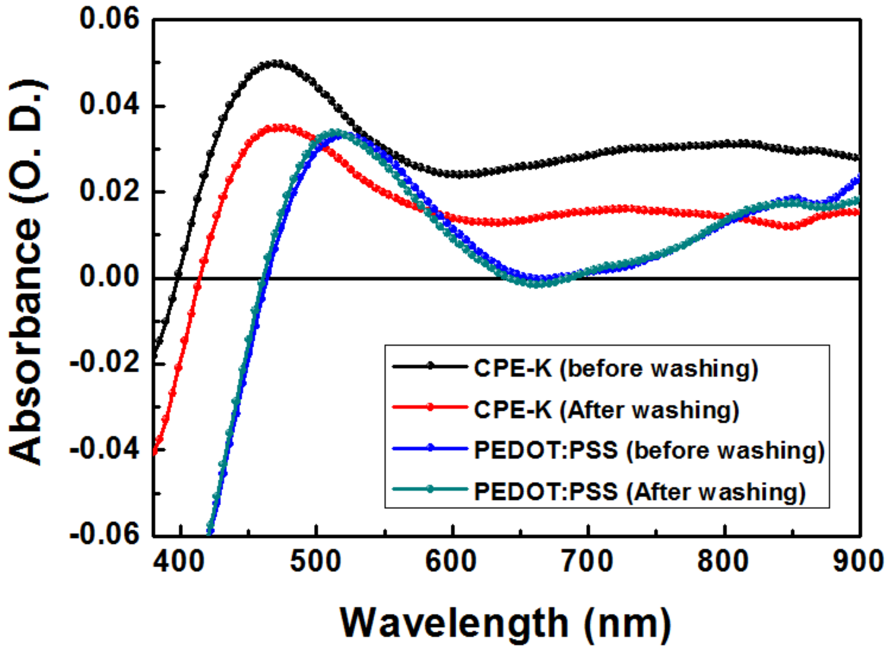

Supplementary Fig. 2. Absorption spectra of PEDOT:PSS and CPE-K films before and after washing with DMF. ITO substrate was used as baseline.

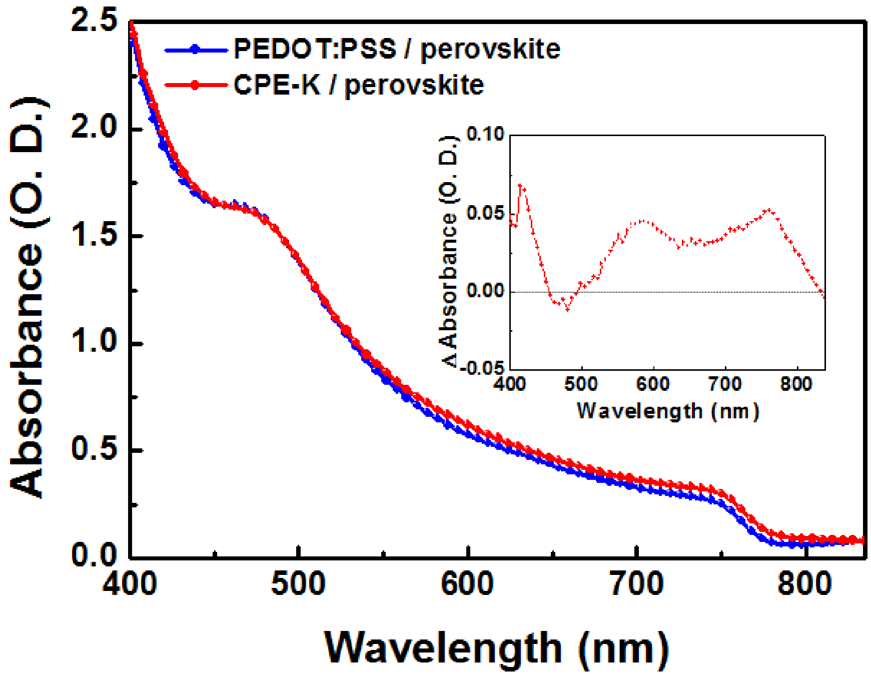

Supplementary Fig. 3. UV-vis absorption spectra of $MAPbI_{3-X}Cl_X$ perovskite films spin-coated on top of PEDOT:PSS and CPE-K. Inset shows absorption difference between them.

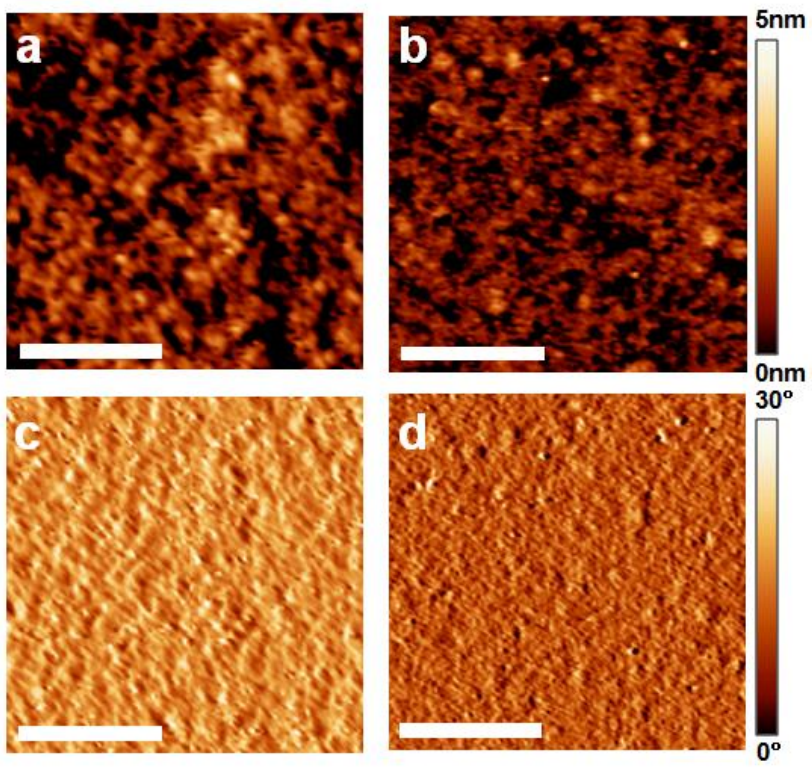

Supplementary Fig. 4. AFM topography (upper row) and phase images (lower row) of PEDOT:PSS (a and c) and CPE-K (b and d), respectively. Scale bar is 1 μm in all AFM images.

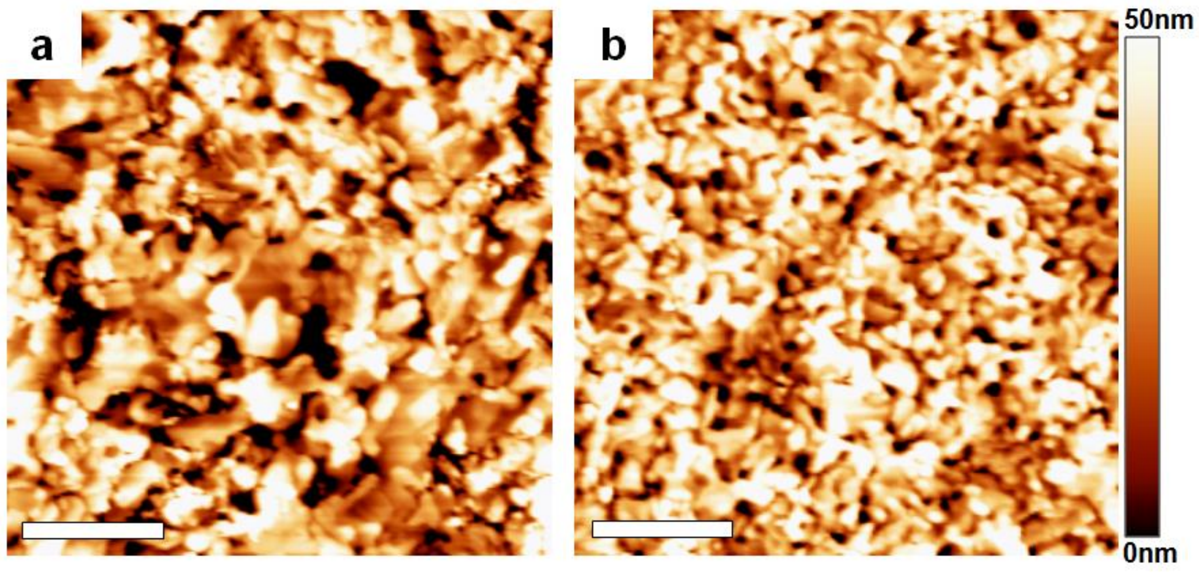

Supplementary Fig. 5. AFM topography images of perovskite films on (a) PEDOT:PSS and (b) CPE-K. Scale bar is 2 μm.

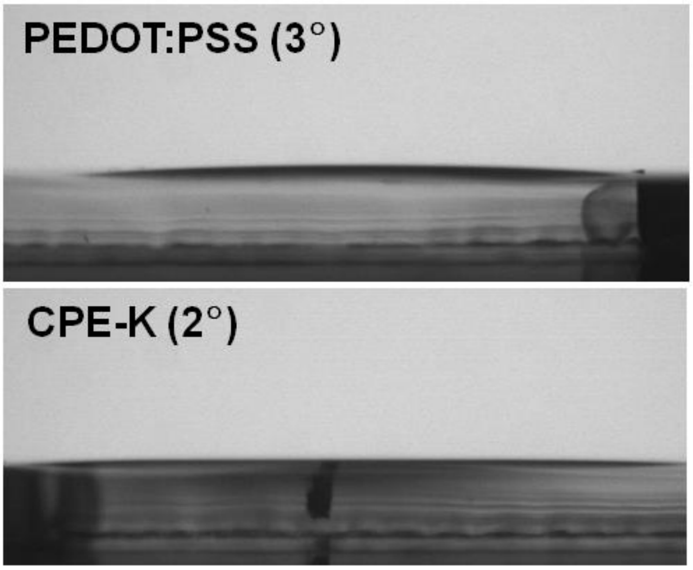

Supplementary Fig. 6. Contact angles of PEDOT:PSS and CPE-K films to DMF.

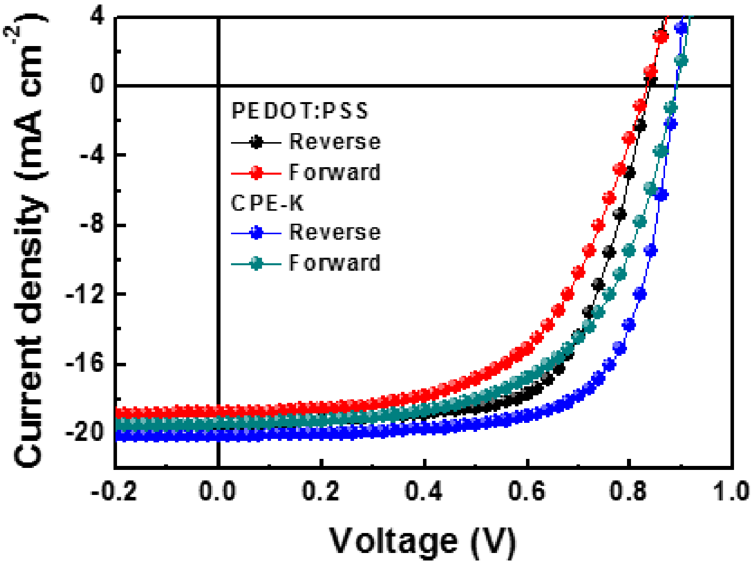

Supplementary Fig. 7. *J-V* curves of pero-SCs with PEDOT:PSS and CPE-K measured by forward and reverse scans with 20 mV voltage steps and 10 ms delay times under AM 1.5G illumination.

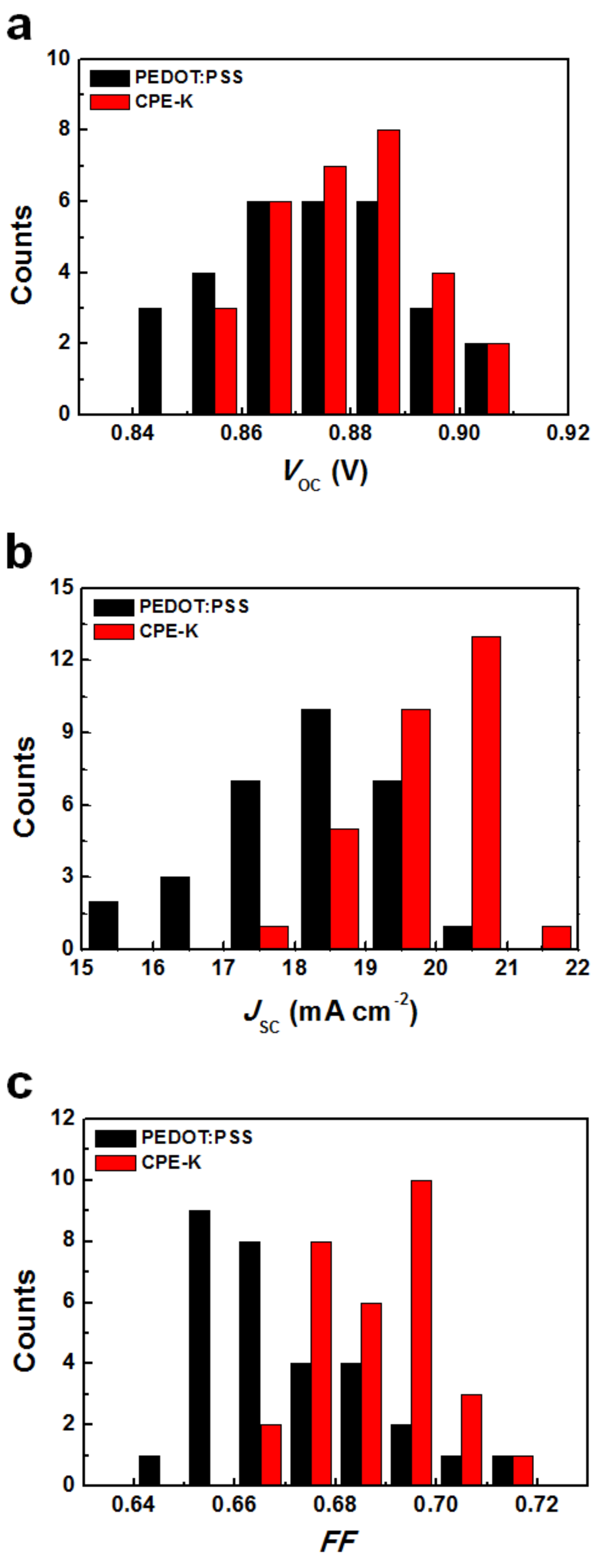

Supplementary Fig. 8. Histograms of device parameters for 30 separate ipero-SCs with PEDOST:PSS and CPE-K. a) J_{SC}, b) V_{OC}, and c) *FF*.

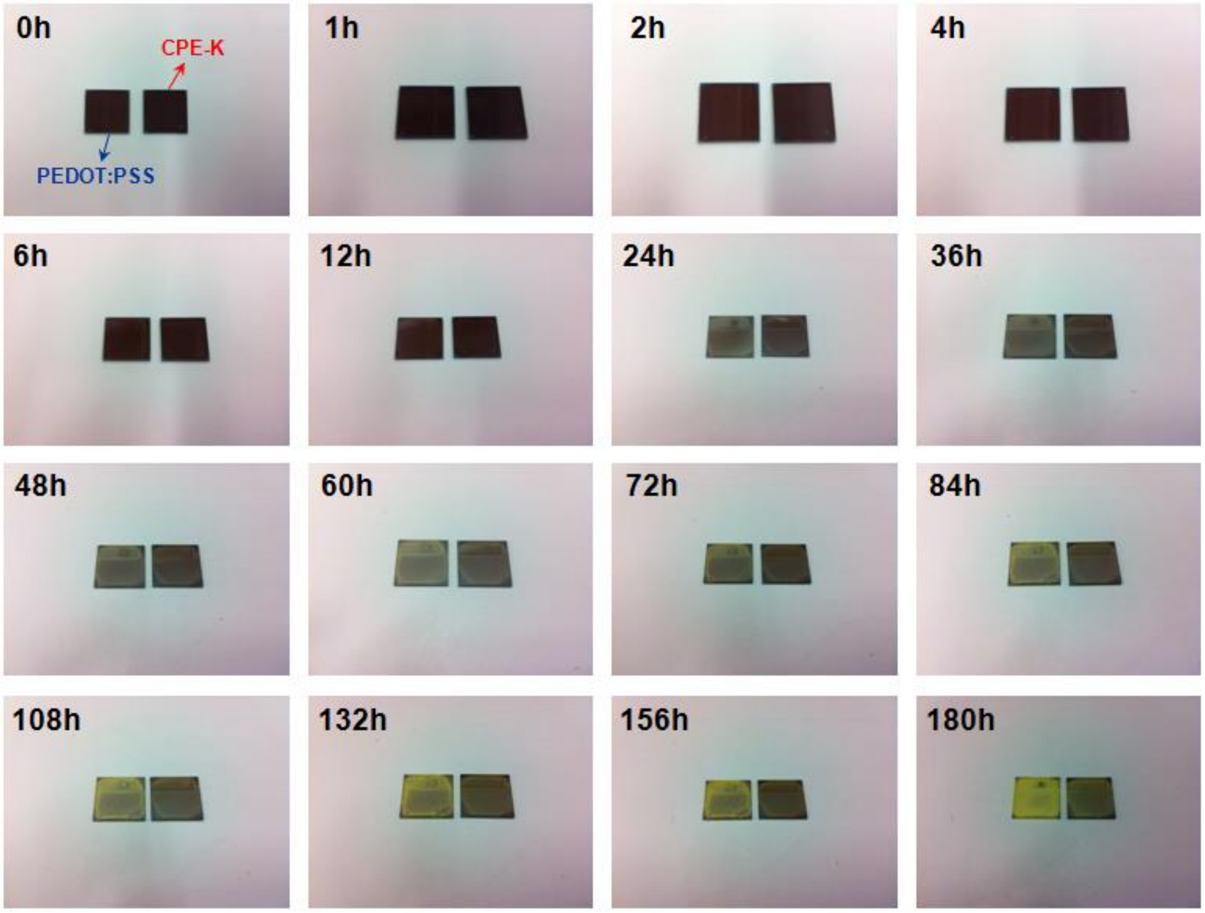

Supplementary Fig. 9. Real images of perovskite films coated on PEDOT:PSS and CPE-K illustrating visible degradation as a function of air exposure time. Average temperature and humidity were 20 ± 3 °C and 40 ± 10% for air stability, respectively.

Supplementary Table 1. Summary of chemical structures, optical, electrochemical properties of various CPEs.

	CPE-K	CPE-Na	CPEPh-Na	PFBT-Na
**Chemical structure**	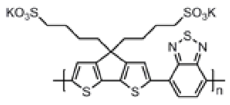 CPE-K	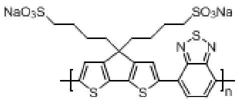 CPE-Na	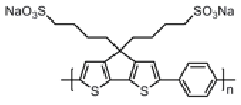 CPEPh-Na	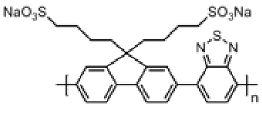 PFBT-Na
**HOMO level (eV)**	4.92	4.92	4.90	5.38
**LUMO level (eV)**	3.54	3.54	2.76	2.94
**Optical bandgap (eV)**	1.38	1.38	2.14	2.44
**Film conductivity (S cm^{-1})**	0.024	0.160	0.022	Not conductive

Supplementary Table 2. Device characteristics of ipero-SCs with different HTL.

HTL	J_{SC} (mA cm^{-2})	V_{OC} (V)	FF	PCE (%)
PEDOT:PSS	19.58	0.84	0.66	10.77
CPE-K	20.10	0.89	0.70	12.51
CPE-Na	16.48	0.84	0.62	8.52
CPEPh-Na	16.97	0.92	0.63	9.77
PFBT-Na	0.04	0.84	0.22	0.01

Supplementary Table 3. Device characteristics of ipero-SCs with PEDOT:PSS and CPE-K measured by forward and reverse scans.

HTL	Scan direction	J_{SC} (mA cm^{-2})	V_{OC} (V)	FF	PCE (%)
PEDOT:PSS	Reverse	19.58	0.84	0.66	10.77
	Forward	18.79	0.83	0.58	9.04
CPE-K	Reverse	20.10	0.89	0.70	12.51
	Forward	19.41	0.89	0.60	10.30

Supplementary Methods

Preparation of perovskite precursor solution.

Lead chloride ($PbCl_2$) was purchased from Sigma-Aldrich and used without purification. Methylammonium iodide (MAI) was synthesized using synthetic routes in previous literature[1]. MAI and $PbCl_2$ with molar ratio of 3:1 were dissolved in *N*,*N*-dimethylformamide (DMF) at concentration of 40 wt.% and this solution was stirred at 60 °C for 6h in nitrogen-filled glovebox.

Film preparation and characterization.

We prepared perovskite films on different substrates for various measurements by using same procedures that were used for optimum devices. SEM images were obtained using FEI XL40 Sirion FEG digital scanning microscope. XRD measurements were carried out using a Bruker, D8 ADVANCE at a scan rate of 2.4° min^{-1}. UV-vis absorption was measured using a OLIS 14 spectrophotometer. AFM images were obtained using a Asylum MFP-3D standard

system AFM microscope in a tapping mode. Contact angle measurements were carried out using DSA 100 (KRUSS, Germany).

Supplementary figure and description

We employed four conjugated polyelectrolytes (CPEs) with different anionic polymer backbones and counter ions. Chemical structures and a variety properties of these CPEs were listed in **Supplementary Table 1**. To compare the effect of different CPEs on device performance, we fabricated ipero-SCs using CPEs as the HTL. **Supplementary Fig. 1a** and **Fig. 1b** show *J*-*V* curves and EQE of the devices as a function of HTLs, respectively. The detailed device parameters are listed in **Supplementary Table 2**. The device with PFBT-Na showed poor device performance, whereas CPE-Na and CPEPh-Na led to comparable PCE to that of the device with PEDOT:PSS. However, the devices with CPE-K exhibited highest PCE of 12.51% among various CPEs. Measured J_{SC} values from *J*-*V* curves were consistent with J_{SC} integrated from EQE curves (**Supplementary Fig. 1b**).

Supplementary Fig. 2 shows absorption spectra of PEDOT:PSS and CPE-K before and after washing them with DMF. DMF is a solvent for dissolving two perovskite precursor materials, MAI and $PbCl_2$. Absorption spectrum of PEDOT:PSS was unchanged after washing with DMF, whereas we observed 30% decrease in optical density (OD) of CPE-K. Although washing process with DMF slightly removed CPE-K film, this layer was still existed on ITO substrate without complete washing out. Absorption spectra of perovskite film on PEDOT:PSS and CPE-K were shown in **Supplementary Fig. 3**. The film on CPE-K exhibited slightly higher OD than that of the film on PEDOT:PSS owing to higher absorption of CPE-K in visible wavelength region. Absorption difference between perovskite films on PEDOT:PSS and CPE-K (**Inset of Supplementary Fig. 3**) was consistent with absorption difference between PEDOT:PSS and CPE-K (**Supplementary Fig. 2**).

To investigate the influence of different substrates on perovskite film morphology, we first performed AFM measurements for PEDOT:PSS and CPE-K. In spite of small differences in topography and phase images, both films exhibited smooth surface with root-mean-square (rms) roughness of 1.0 nm (**Supplementary Fig. 4**). However, different morphology was clearly seen in perovskite films spin-coated on PEDOT:PSS and CPE-K (**Supplementary Fig. 5**). Perovskite film on CPE-K was uniform with complete surface coverage and rms roughness of 14.7 nm (**Supplementary Fig. 5a**), whereas film on PEDOT:PSS exhibited uneven surface with rms roughness of 15.6 nm and large number of voids between crystal boundaries (**Supplementary Fig. 5b**). This implies that CPE-K results in more uniform perovskite film with higher surface coverage than PEDOT:PSS. We also studied surface energy of PEDOT:PSS and CPE-K by performing contact angle measurements. Both film exhibited extremely low contact angles to DMF below 3° (**Supplementary Fig. 6**), indicating that super-hydrophilic surfaces of both PEDOT:PSS and CPE-K films are compatible with perovskite precursor solution and thus enable successful formation of perovskite film on them.

For PL decay transients, we prepared three types of samples (glass/perovskite, glass /PEDOT:PSS/perovskite, and glass/CPE-K/perovskite) using perovskite film with optimum thickness (250±20 nm) for best device performance. We excited samples at 405 nm and collected PL decay transients at 770 nm. Perovskite film on CPE-K exhibited faster average PL decay time (1.41 ns) compared to those of films on bare glass and PEDOT:PSS (Bare glass: 153 ns and PEDOT:PSS: 91 ns). These values are in good agreement with PL lifetimes for $MAPbI_{3-X}Cl_X$ perovskite film reported previously[2]. PL decay time measurement reveals that CPE-K has superior capability for hole transport from perovskite light absorber to ITO anode.

Supplementary Fig. 7 exhibits *J-V* characteristics of ipero-SCs with PEDOT:PSS and

CPE-K measured by forward and reverse scan. The detailed device parameters were listed in **Supplementary Table 3**. We performed scans with 20 mV voltage steps and 10ms delay times at 100 mW cm^{-2} irradiation. There was slight hysteresis in both devices which may result from interfacial traps induced in HTL or ferroelectric property of perovskite.

Supplementary Fig. 8 shows distribution histograms of three solar cell parameters for 30 separate devices with PEDOT:PSS and CPE-K. Both devices had similar V_{OC} values (0.84-0.91 V) (**Fig. S8a**), whereas the devices with CPE-K exhibited higher J_{SC} and FF than those of the devices with PEDOT:PSS (**Supplementary Fig. 8b** and **8c**). As a result, the device with CPE-K yielded higher average PCE (11.20%) than that of the device with PEDOT:PSS (9.37%). This high device efficiency was attributed to improved perovskite film morphology (**Fig. 2**) and efficient hole transport from perovskite to ITO anode (**Fig. 3**) by CPE-K HTL.

Supplementary Fig. 9 presents visible degradation of perovskite films on PEDOT:PSS and CPE-K as a function of air exposure time. We tested film stability in ambient air condition with average temperature of 20±3° and humidity of 40±10%. We observed the same dark-brown films on both PEDOT:PSS and CPE-K. After 24 h, although visible degradation occurred in the film on CPE-K, degradation rate of film on PEDOT:PSS was much faster than that of film on CPE-K. After 180 h, perovskite film on CPE-K still maintained dark brown color, whereas film on PEDOT:PSS was completely changed to yellow solid, indicating decomposition of perovskite phase into PbI_2 by the presence of water[3]. These results reveal that acidic nature of PEDOT:PSS has bad influence on perovskite film stability and thus is detrimental for device stability in air condition (**Fig. 5d**).

Supplementary References

1. Jeng, J.-Y. *et al.* CH3NH3PbI3 Perovskite/Fullerene Planar-Heterojunction Hybrid Solar Cells. *Adv. Mater.* **25**, 3727-3732 (2013).

2. Stranks, S. D. *et al.* Electron-Hole Diffusion Lengths Exceeding 1 Micrometer in an Organometal Trihalide Perovskite Absorber. *Science* **342**, 341-344 (2013).

3. Frost, J. M. *et al.* Atomistic Origins of High-Performance in Hybrid Halide Perovskite Solar Cells. *Nano Lett.* **14**, 2584-2590 (2014).
